# Population structure and antimicrobial susceptibility of *Pseudomonas aeruginosa* from animal infections in France

**DOI:** 10.1186/s12917-015-0324-x

**Published:** 2015-01-21

**Authors:** Marisa Haenni, Didier Hocquet, Cécile Ponsin, Pascal Cholley, Christophe Guyeux, Jean-Yves Madec, Xavier Bertrand

**Affiliations:** Agence Nationale de Sécurité Sanitaire (Anses), Unité Antibiorésistance et Virulence Bactériennes, Lyon, France; Laboratoire d’Hygiène Hospitalière, UMR 6249 CNRS Chrono-environnement, Université de Franche-Comté, Centre Hospitalier Universitaire, Besançon, France; Département DISC, Institut FEMTO-ST, UMR 6174 CNRS, Université de Franche-Comté, Belfort Cedex, France

**Keywords:** *Pseudomonas aeruginosa*, Animal, MLST, Antimicrobial susceptibility

## Abstract

**Background:**

*Pseudomonas aeruginosa* is a major human pathogen, which also affects animals. It is thought that *P. aeruginosa* has a non-clonal epidemic population structure, with distinct isolates found in humans, animals or the environment. However, very little is known about the structure of the *P. aeruginosa* population from diseased animals. Data on antimicrobial resistance are also scarce.

**Results:**

Thirty-four already registered and 19 new MLST profiles were identified. Interestingly, a few clones were more prevalent, and clones associated to human outbreaks were also detected. Multidrug resistance phenotypes were overall rare.

**Conclusion:**

We highlight the non clonal structure of the population and show a higher prevalence of specific clones, possibly correlating with higher pathogenicity. The low proportion of antimicrobial resistance contrasts with the high resistance rate of human isolates.

**Electronic supplementary material:**

The online version of this article (doi:10.1186/s12917-015-0324-x) contains supplementary material, which is available to authorized users.

## Background

*Pseudomonas aeruginosa* is a major opportunistic human pathogen, causing infections mostly in patients with impaired immune systems [1], or in people suffering from cystic fibrosis. It is also a cause of diseases in both livestock and companion animals, including otitis and urinary tract infections in dogs, mastitis in dairy cows, endometritis in horses and hemorrhagic pneumoniae in fur-bearing animals such as mink or foxes [2-4].

*P. aeruginosa* is naturally resistant to many classes of drugs and its capacity to rapidly acquire resistance during treatment is a frequent source of therapeutic failures in humans [5]. It is generally admitted that the *P. aeruginosa* population is non clonal, i.e. that population structure of *P. aeruginosa* is highly diverse with a high number of clonal groups with no or poor association between clonal groups and ecological niches or specific diseases. Moreover, human and environmental isolates are distinct, and there are no specific clones associated with a specific habitat, disease or animal species, except for a few multi-drug resistant clusters (called ‘high-risks clones’) that are spreading worldwide [6,7].

However, since the structure of the *P. aeruginosa* population from animals remains unknown in France, the objective of this study was to provide a snapshot on the genetic diversity of this bacterium, to be compared with clones circulating in humans. In line with a possible risk of animal-to-human transfer, susceptibility of those isolates to antimicrobials used in human medicine was also investigated.

## Results

A total of 68 isolates was genotyped by PFGE and 60 different pulsotypes (PTs) were obtained (see Additional file 1). The eight very close PTs were shared by two or three animals that had no epidemiological link and originated from diverse locations in France.

Since PFGE was proved to be more discriminatory than MLST, we considered that isolates with a very close PT shared the same ST [8]. MLST was thus performed on one isolate representative of each of the 60 PTs, leading to the identification of 53 distinct STs (http://pubmlst.org/paeruginosa). Among them, 34 were already registered in the MLST database while 19 were new (ST1611-1614 and ST1708-1722). ST108, which was represented by very close PTs in two dogs (24264/25810), was additionally found in a cow and a horse (26849/26185; see Additional file 2) presenting divergent PTs. ST395 and ST1614 were represented by 3 isolates, whereas 6 STs included 2 isolates (see Additional file 2). The 38 remaining STs were represented by a single isolate. Three STs, including two new profiles, were recovered in both dogs and horses (ST155, ST1611 and ST1614).

**Figure 1:**
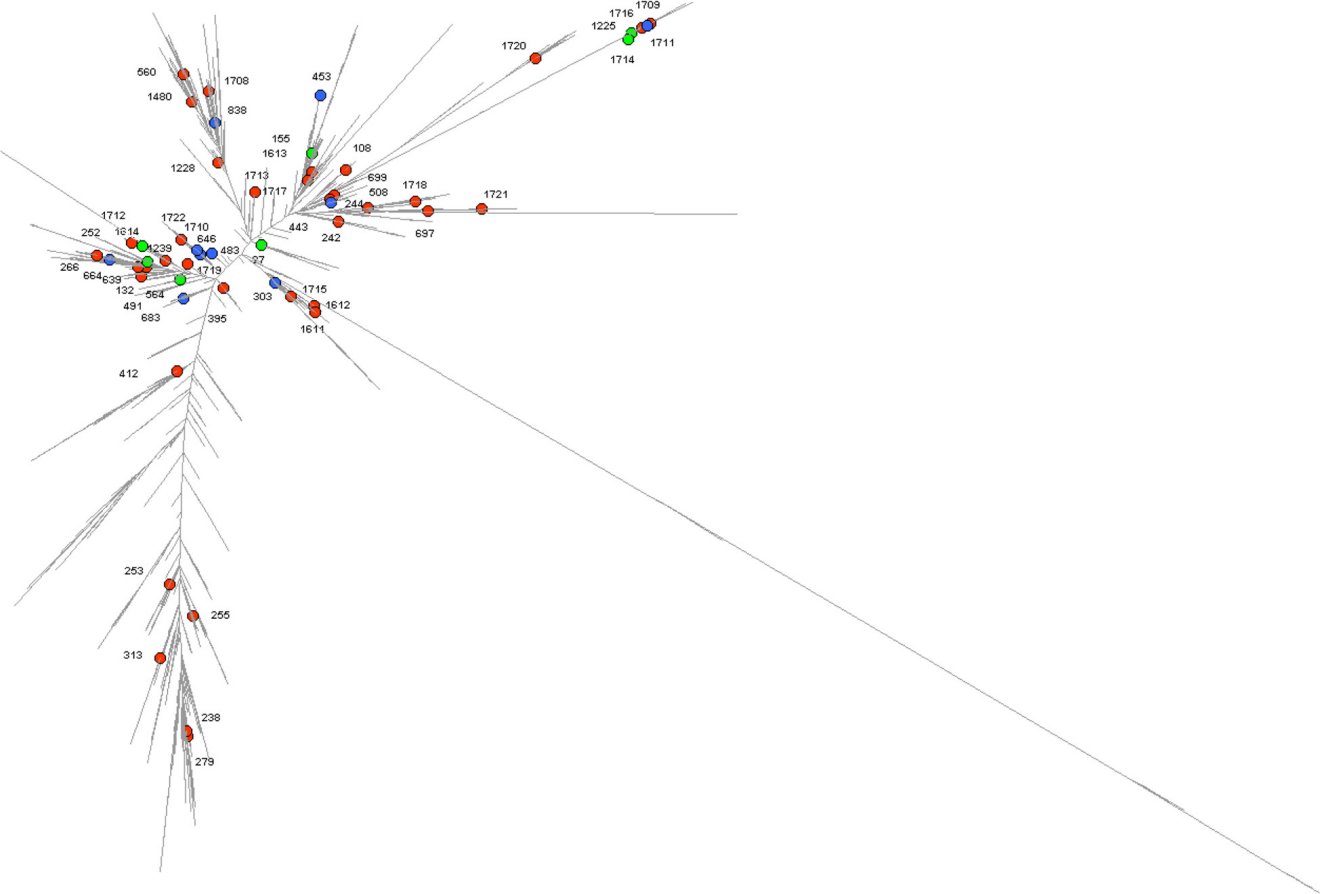
**Comparative analysis based on concatenated sequences of the seven housekeeping genes of** ***P. aeruginosa*** **MLST scheme (n = 1722).** The STs found in dogs, bovines, and horses in the present collection are represented with red, blue, and green spots, respectively.

We then determined whether the clones of *P. aeruginosa* found in animals in France clustered on the global dendrogram of the 1740 currently defined STs (Figure 1). The maximum likelihood (ML) tree produced by the alignment of the concatenated gene sequences of the currently known STs displayed a bush-like structure showing the non-clonal structure of the species [9]. As illustrated by Figure 1, the 53 STs of our collection scattered throughout the ML tree.

Finally, we investigated the antimicrobial susceptibility of all *P. aeruginosa* isolates. Susceptibilities to fosfomycin, ciprofloxacin and gentamicin were the most frequently affected, with 54.4%, 42.6%, and 41.2% of resistant isolates, respectively (Table 1). Resistant isolates mostly came from dogs and significant differences (p < 0.05) in resistances compared to horses and cows were observed for ticarcillin – clavulanic acid, gentamicin and ciprofloxacin. Multidrug resistance (non-susceptible to three or four antimicrobial categories) was absent in dairy cows and horses, whereas it was relatively common (16/46; 35%) in dog isolates (Additional file 2). Among these isolates, combinations of resistances to β-lactams-aminoglycosides-fosfomycin or to β-lactams-aminoglycosides-ciprofloxacin were the most frequently detected. No resistance to carbapenems was observed.

**Table 1:** **Antimicrobial resistance of the 68** ***P. aeruginosa*** **strains**

**Antibiotics**	**Breakpoints (mm; S≥/R<)**	**Horses and cows (n = 22)**		**Dogs (n = 46)**		**Comparison of proportions between dogs and other animals**
		**Number of resistant strains**	**Percentage of resistant strains (%)**	**Number of resistant strains**	**Percentage of resistant strains (%)**	**p**
Piperacillin	18/18	0	0.0	0	0.0	.
Piperacillin-tazobactam	19/19	0	0.0	0	0.0	.
Ticarcillin	22/22	3	13.6	11	23.9	0.5093
Ticarcillin-clavulanic acid	22/22	1	4.5	16	34.8	0.0071
Ceftazidime	19/19	0	0.0	0	0.0	.
Cefepime	19/19	0	0.0	4	8.7	0.3817
Aztreonam	27/19	0	0.0	3	6.5	NA^1^
Imipenem	22/17	0	0.0	0	0.0	.
Meropenem	22/15	0	0.0	0	0.0	.
Amikacin	17/15	0	0.0	7	15.2	0.1322
Gentamicin	16/16	2	9.1	26	56.5	0.0002
Tobramycin	16/16	1	4.5	5	10.9	0.6868
Fosfomycin	14/14	15	68.2	22	47.8	0.1149
Ciprofloxacin	25/22	0	0.0	29	63.0	<0.0001

^1^NA: not applicable.

## Discussion

In veterinary medicine, *P. aeruginosa* is not a widespread pathogen but is responsible for a variety of difficult-to-treat infections. Our study adds data to the limited comprehensive picture of the population structure of clinical *P. aeruginosa* from animals [6,7], and highlights a high genetic diversity even though 8 clones were surprisingly not unique. Both indistinguishable and very close PFGE profiles were also found among mink *P. aeruginosa* from different regions in Eastern China [10], suggesting that certain *P. aeruginosa* clones, similarly to humans, would be more pathogenic to animals than others. These clones may spread either through a food or water supply, or may persist in the environment, as demonstrated for *P. aeruginosa* infecting mink [4,10].

STs were also diverse with numerous new profiles (19/53). Their scattering throughout the ML tree indicates a poor association between an animal species and a specific subset of the *P. aeruginosa* population. Kidd *et al.* drew similar conclusions from eBURST data and from the calculation of genetic diversity index on animal isolates recovered in Queensland (Australia) [6]. Additionally, we identified five STs that are internationally widespread clones associated to human outbreaks and sometimes to multi-drug resistance phenotypes (ST27, ST155, ST253, ST395, ST560). These specific clones were found in nine dogs (ST395, n = 3; ST560, n = 3; ST253, n = 2; ST155, n = 1) and two horses (ST27, n = 1; ST155, n = 1). However, only one of these animal isolates, belonging to ST395, presented a multidrug resistant phenotype (see below and Additional file 2). Seven STs (ST155, ST242, ST244, ST253, ST266, ST508 and ST560) reported in this study were previously recovered in animals in South East Queensland, Australia [6]. On the contrary, two STs (ST863 and ST883) responsible for outbreaks in Australian horses were not retrieved in our collection [2].

Antimicrobial resistance was principally observed for fosfomycin, ciprofloxacin and gentamicin, a situation that may be explained by the frequent veterinary use of aminoglycosides and fluoroquinolones in France, especially for dog otitis. However, the high proportion of fosfomycin-resistant isolates cannot be attributed to the use of this antibiotic, which is not authorized in veterinary medicine. Multidrug patterns were only detected in dogs, possibly due to the fact that pets are more treated than horses and cows, or that these animals may have been sampled after a first treatment failure.

These data may differ from other reports in different animal species and countries [10-14], even though comparing resistance rates obtained with different standards and sampling strategies is of limited value. Of note, no carbapenem resistance was observed here, contrary to a recent report from a dog in China, which suggested a human-to-dog transfer [15]. Notably, the most frequent epidemic clones associated to multidrug resistance in humans, also called ‘high-risk clones’, *i.e.* ST111, ST235 and ST175, were not recovered in our collection [8].

## Conclusion

*P. aeruginosa* is a major human pathogen that is still poorly documented in the veterinary field. Here, we confirm the non-clonal epidemic structure of the *P. aeruginosa* population and suggest a poor association between an animal species and a specific clone, even though certain clones seem to be more prevalent than others. We found a low proportion of drug resistant *P. aeruginosa* in diseased cows and horses, except for fosfomycin. Resistance phenotypes were much more frequent in dogs, and multidrug resistant *P. aeruginosa* seem to emerge mainly in those suffering from otitis. However, such multidrug phenotypes are still rare in other animal species which, at this stage, contrasts with the high resistance rates observed in human clinical isolates, notably to β-lactams [16]. Nevertheless, antimicrobial resistance in animal *P. aeruginosa* should be closely monitored in the future, in line with possible animal-to-human transfers between pets and owners. This may be especially important for patients with cystic fibrosis, which often results from unique *P. aeruginosa* strains acquired in the environment of the patient.

## Methods

### Isolate collection

Between 2008 and 2011, we randomly collected *P. aeruginosa* through the Resapath, the long-term surveillance network for antimicrobial resistance in pathogenic bacteria in France (www.resapath.anses.fr). A total of 68 consecutive and non-duplicate clinical isolates of *P. aeruginosa* from dogs (n = 46), dairy cows (n = 12) and horses (n = 10) were recovered. These isolates were mostly recovered from otitis in dogs and mastitis in cattle (Additional file 2), and were epidemiologically non-related since they came from different animals, different families/farms, presented different sampling dates and/or originated from different French departments. Isolates were identified by matrix-assisted laser desorption ionization-time of flight mass spectrometry (MALDI-TOF MS) with a Microflex LT (Bruker Daltonik GmbH, Bremen, Germany), according to the manufacturer’s procedure.

### Genotyping

The macrorestriction (using *Dra*I) profile of total DNA from each isolate was determined by Pulsed-Field Gel Electrophoresis (PFGE), as previously described [5]. GelCompar software was used for cluster analysis (Applied Maths, Kortrijk, Belgium). The Dice correlation coefficients were grouped and the UPGMA clustering algorithm was used to depict the groups as a dendrogram. Pulsotypes (PTs) were defined according to international recommendations [17]. Multi Locus Sequence Typing (MLST) was performed according to the protocol of Curran *et al.* modified by van Mansfeld *et al.* [18]*.* Nucleotide sequences were determined for internal fragments of the *acsA*, *aroE*, *guaA*, *mutL*, *nuoD*, *ppsA,* and *trpE* genes, on both strands, and were compared with sequences in the *P. aeruginosa* MLST website (http://pubmlst.org/paeruginosa) for the assignment of allele numbers and sequence types (ST). Clonal complexes (CCs) are defined as a group of STs sharing at least 5 loci, using the START2 software. When two isolates presented very close PTs (according to Tenover’s criteria), only the MLST profile of one representative isolates was determined and then inferred to the other isolates sharing the same PT.

### MLST analysis

In order to build a dendrogram with the 1740 STs available at the time of the study (including the new STs described in this collection), we concatenated the sequences of 7 MLST genes to form a 2,882-bp sequences alignment. We used the *Pseudomonas fluorescens* Pf0-1 as the outgroup strain [19]. The best-fit nucleotide substitution model for this data was GTR + G + I, as determined with jModelTest 0.1.1. Maximum likelihood tree was constructed with RAxML 7.2.8. and visualized with Dendroscope 3.2.10. In every case, 1000 bootstrap repetitions gave values above 900 for most branches.

### Antimicrobial susceptibility testing

Susceptibility testing was performed by the disc diffusion method according to the guidelines of the Antibiogram Committee of the French Society for Microbiology (CA-SFM; www.sfm-microbiologie.fr). Susceptibility to the following 14 antibiotics of human interest was tested: piperacillin (75 μg), piperacillin + tazobactam (75/10 μg), ticarcillin (75 μg), ticarcillin + clavulanic acid (75/10 μg), ceftazidime (30 μg), cefepime (30 μg), imipenem (10 μg), meropenem (10 μg), aztreonam (30 μg), amikacin (30 μg), gentamicin (15 μg), tobramycin (10 μg), fosfomycin (50 μg), ciprofloxacin (5 μg). Bacteria were classified as susceptible, intermediate or resistant according to the recommendations and the human-specific clinical breakpoints approved by the French Society for Microbiology*. E. coli* ATCC 25922 and *P. aeruginosa* ATCC 27853 were used as quality controls.

### Statistics

Comparisons of proportions were done using the Chi-2 test (with Yate’s correction when needed), and the significance level was set to 0.05.

## Additional files

Additional file 1:
**Epidemiological data and resistance profiles of all 68 tested isolates.** Additional file 1 includes a detailed description of resistance profiles, sequence types and epidemiological data (sampling data, geographical origin and pathology) of all studied isolates. Additional file 2:
**Pulsed-Field Gel Electrophoresis (PFGE) profile of all** ***P. aeruginosa*** **isolates.** Additional file 2 presents the comparison of PFGE profiles of all 68 studied strains.
