# Supplementary material for: Population structure and antimicrobial susceptibility of Pseudomonas aeruginosa from animal infections in France
Source: BMC Vet Res. 2015 Jan 21;11:9. doi: 10.1186/s12917-015-0324-x (PMC4307146; doi:10.1186/s12917-015-0324-x)
Supplement: Additional file 1: — Epidemiological data and resistance profiles of all 68 tested isolates. Additional file 1 includes a detailed description of resistance profiles, sequence types and epidemiological data (sampling data, geographical origin and pathology) of all studied isolates. [file 12917_2015_324_MOESM1_ESM.pdf]

Table S1. Epidemiological data and resistance profiles of all 68 tested isolates

Strain	Geographical origin	Sampling date	Animal	Pathology	Resistance profile[3]	ST
25353	Alpes-Maritimes	15.05.2008	Dog	Otitis	GEN	491
25355	Alpes-Maritimes	04.06.2008	Dog	Otitis	FOS, CIP	639
24220	Loire-Atlanntique	03.01.2009	Dog	Otitis	TIC, TTC, CIP	266
24104	Eure-et-Loire	13.01.2009	Dog	Otitis	FEP, GEN, FOS*	1611
24254	Tarn	20.01.2009	Dog	Metritis	TTC, GEN, FOS, CIP*	1612
24969	Vosges	11.03.2009	Dog	Otitis	TIC, GEN, CIP*	1720
24257	Hérault	27.04.2009	Dog	Otitis	FEP, GEN, FOS, CIP*	255
24258	Hérault	17.06.2009	Dog	Otitis	FOS, CIP	1718
24108	Oise	14.07.2009	Dog	Otitis	GEN, FOS	**560**[4]
24260	Hérault	27.07.2009	Dog	NP[2]	TTC, CIP	238
25363	Alpes-Maritimes	07.10.2009	Dog	Otitis	FOS, CIP	1708
25369	Alpes-Maritimes	08.10.2009	Dog	Otitis	FOS, CIP	508
25372	Alpes-Maritimes	04.11.2009	Dog	Otitis	AMI, GEN	242
25361	Alpes-Maritimes	12.11.2009	Dog	Otitis	TIC, TTC, FOS, CIP*	1715
24692	Gard	22.12.2009	Dog	Otitis	GEN	**253**
24117	Alpes-Maritimes	15.01.2010	Dog	Otitis	TTC, FEP, GEN, CIP*	1709
24325	Savoie	22.01.2010	Dog	Otitis	TIC, TTC, AZT, AMI, GEN, TOB, CIP*	1721
24264	Hérault	29.01.2010	Dog	Otitis	CIP	108
24972	Meurthe-et-Moselle	01.02.2010	Dog	Otitis	TTC, GEN, FOS*	1613
24974	Moselle	01.03.2010	Dog	Otitis	AMI, GEN, TOB, FOS	1228
24268	Alpes-Maritimes	09.03.2010	Dog	Otitis	FOS	699
25055	Gard	22.03.2010	Dog	Otitis	GEN, FOS, CIP*	443
25262	Savoie	19.04.2010	Dog	Skin infection	TIC, TTC, GEN	1722
25324	Val-de-Marne	23.04.2010	Dog	Otitis	GEN	313
25320	Val-de-Marne	27.04.2010	Dog	Otitis	AMI, GEN, TOB, FOS, CIP*	1717
25322	Val-de-Marne	29.04.2010	Dog	Otitis	AMI, GEN, TOB, FOS, CIP*	1713
25270	Val-de-Marne	03.05.2010	Dog	Urinary infection	CIP	1239
25272	Val-de-Marne	04.05.2010	Dog	Otitis	TIC, GEN, CIP*	1614
25293	Val-de-Marne	01.06.2010	Dog	Otitis	GEN, CIP	**253**
25331	Val-de-Marne	10.06.2010	Dog	Otitis	CIP	**395**

25303	Val-de-Marne	15.06.2010	Dog	NP	TTC, FEP, GEN	1716
25292	Val-de-Marne	24.06.2010	Dog	Otitis	TTC, AMI, GEN, FOS*	1480
25163	Alpes-Maritimes	29.06.2010	Dog	Skin infection	CIP	**155**
25187	Nièvre	10.07.2010	Dog	Otitis	GEN	279
25305	Val-de-Marne	23.07.2010	Dog	NP	TIC, TTC, FOS, CIP*	443
25827	Val-de-Marne	28.07.2010	Dog	Skin infection	TIC, TTC, GEN, FOS, CIP*	**395**
25189	Val-de-Marne	17.08.2010	Dog	Otitis	TIC, TTC, CIP	132
25319	Cantal	25.08.2010	Dog	Otitis	TTC, GEN	1712
25810	Val-de-Marne	09.09.2010	Dog	Otitis	CIP	108
25812	Val-de-Marne	09.09.2010	Dog	Otitis	GEN, FOS, CIP*	697
25799	Val-de-Marne	16.09.2010	Dog	Otitis	CIP	639
25746	Val-de-Marne	28.09.2010	Dog	Bone infection	TIC, TTC, AZT, FOS	412
25751	Val-de-Marne	08.10.2010	Dog	Otitis	AMI, GEN, TOB	1719
25752	Val-de-Marne	09.10.2010	Dog	Skin infection	CIP	**560**
25745	Val-de-Marne	25.10.2010	Dog	NP	TIC, TTC, AZT, FOS	**560**
26008	Haut-Rhin	24.11.2010	Dog	Otitis	FOS, CIP	**395**
24362	Moselle	02.10.2009	Bovine	Respiratory infection	GEN, FOS	683
24970	Doubs	21.01.2010	Bovine	Digestive infection	FOS	453
25119	Maine-etl-Loire	22.04.2010	Bovine	Mastitis	TIC, FOS	244
26858	Manche	08.06.2010	Bovine	Mastitis	TIC	483
26006	Meurthe-et-Moselle	01.07.2010	Bovine	Mastitis	FOS	838
26849	Lozère	09.07.2010	Bovine	Mastitis	FOS	108
25574	Yonne	13.07.2010	Bovine	Mastitis	FOS	683
25919	Jura	21.09.2010	Bovine	Mastitis	none	303
26317	Isère	24.11.2010	Bovine	Mastitis	FOS	664
26119	Savoie	21.12.2010	Bovine	Respiratory infection	none	1711
26374	Cantal	06.01.2011	Bovine	Respiratory infection	FOS	1710
26319	Nièvre	07.01.2011	Bovine	Respiratory infection	TIC, TTC, FOS	646
24466	Hérault	05.11.2009	Horse	Skin infection	FOS	1225
25685	Hautes-Alpes	17.05.2010	Horse	Eye infection	none	1614
26861	Manche	16.07.2010	Horse	Metritis	FOS	**27**
26007	Meurthe-et-Moselle	01.08.2010	Horse	Skin infection	FOS	564
26854	Bas-Rhin	01.02.2011	Horse	NP	none	1614
26185	Haute-Savoie	11.02.2011	Horse	NP	GEN, TOB	108

26973	Mayenne	28.03.2011	Horse	Respiratory infection	FOS	1714
26863	Manche	06.07.2011	Horse	Metritis	FOS	252
27641	Val-de-Marne	30.08.2011	Horse	Respiratory infection	none	**155**
27627	Haute-Savoie	07.09.2011	Horse	Skin infection	FOS	1611

[1] underlined: strains with the same pulsotype (25272/25685/26854; 24104/27627; 24692/25293; 24264/25810; 25055/25305; 24108/25752; 25827/26008)

[2] NP, not provided

[3] TIC, ticarcillin; TTC, ticarcillin – clavulanic acid; FEP, cefepim; ATM, aztreonam; AMI, amikacin; GEN, gentamicin; TOB, tobramycin; FOS, fosfomycin; CIP, ciprofloxacin

[4] in bold: STs associated to human outbreaks and sometimes to multidrug resistant phenotypes.

* Isolates presenting a multidrug resistant phenotype (resistance to 3 or 4 antibiotic families)
