# Supplementary figures and images for: Population structure and antimicrobial susceptibility of Pseudomonas aeruginosa from animal infections in France
Source: BMC Vet Res. 2015 Jan 21;11:9. doi: 10.1186/s12917-015-0324-x (PMC4307146; doi:10.1186/s12917-015-0324-x)

**Figure S1.** PFGE profile of all *P. aeruginosa* isolates.

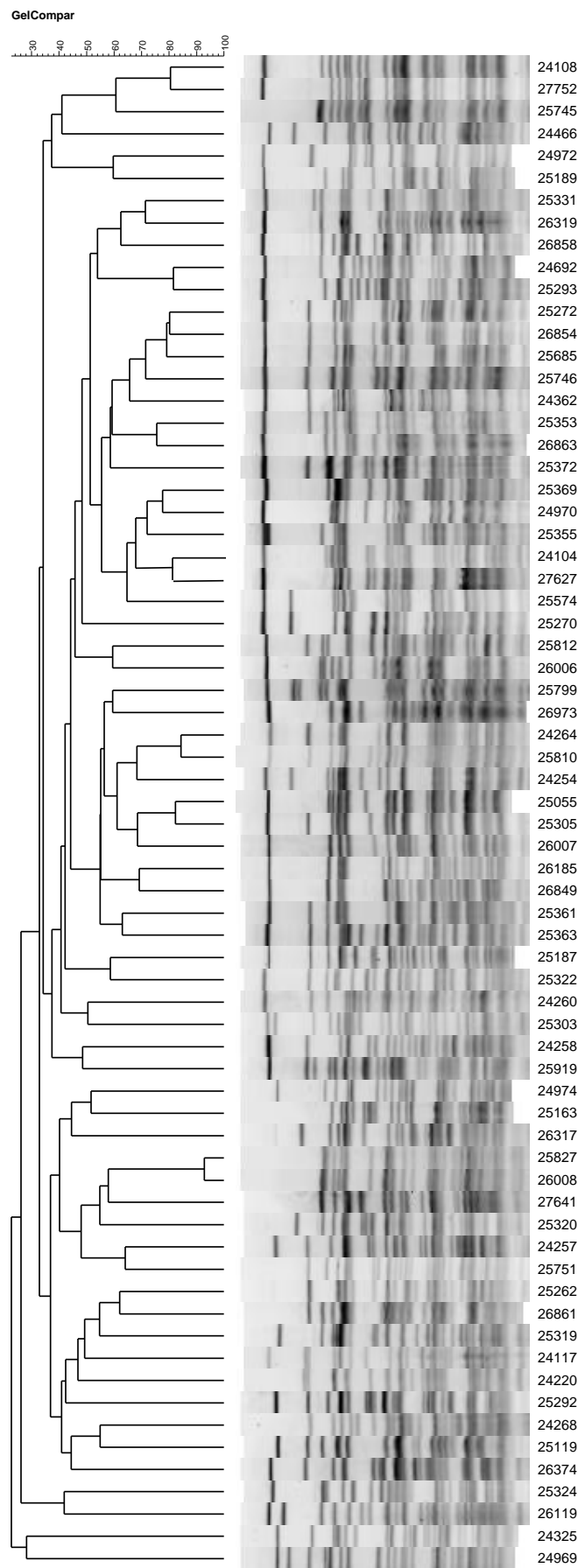

Supplement: Additional file 2: — Pulsed-Field Gel Electrophoresis (PFGE) profile of all P. aeruginosa isolates. Additional file 2 presents the comparison of PFGE profiles of all 68 studied strains. [file 12917_2015_324_MOESM2_ESM.pdf]
